# Addressing SARS-CoV-2 evolution: neutralization of emerging variants of concern by the AVX/COVID-12 ‘Patria’ vaccine based on HexaPro-S ancestral Wuhan spike or its updated BA.2.75.2 version

**DOI:** 10.3389/fimmu.2025.1565934

**Published:** 2025-05-19

**Authors:** Gregorio Carballo-Uicab, Gabriela Mellado-Sánchez, Edith González-González, Juana Salinas-Trujano, Ivette Mendoza-Salazar, Karina López-Olvera, Keyla M. Gómez-Castellano, Ma. Isabel Salazar, Jesús M. Torres-Flores, Héctor Elías Chagoya-Cortés, Georgina Paz-De la Rosa, Ignacio Mena, Oscar Rojas-Martínez, Jesús Horacio Lara-Puente, Gustavo Javier Peralta-Sánchez, David Sarfati-Mizrahi, Alejandro Torres-Flores, Weina Sun, Florian Krammer, Adolfo García-Sastre, Peter Palese, Constantino López-Macías, Bernardo Lozano-Dubernard, Sonia M. Pérez-Tapia, Juan C. Almagro

**Affiliations:** ^1^ Unidad de Desarrollo e Investigación en Bioterapéuticos (UDIBI), Escuela Nacional de Ciencias Biológicas, Instituto Politécnico Nacional, México City, México; ^2^ Laboratorio Nacional Para Servicios Especializados de Investigación, Desarrollo e Innovación (I+D+i) Para Farmoquímicos y Biotecnológicos, LANSEIDI-FarBiotec-CONACyT, México City, México; ^3^ Departamento de Inmunología, Escuela Nacional de Ciencias Biológicas, Instituto Politécnico Nacional (ENCB-IPN), México City, México; ^4^ Laboratorio Nacional de Vacunología y Virus Tropicales (LNVyVT), Escuela Nacional de Ciencias Biológicas, Instituto Politécnico Nacional, México City, México; ^5^ Consultora Mextrategy, S.A.S. de C.V., México City, México; ^6^ Laboratorio Avi-Mex, S.A. de C.V. (Avimex), México City, México; ^7^ Unidad de Investigación Médica en Inmunoquímica, UMAE Hospital de Especialidades, Centro Médico Nacional Siglo XXI, Instituto Mexicano del Seguro Social (IMSS), México City, México; ^8^ Department of Microbiology, Icahn School of Medicine at Mount Sinai, New York, NY, United States; ^9^ Department of Pathology, Molecular and Cell-Based Medicine, Icahn School of Medicine at Mount Sinai, New York, NY, United States; ^10^ Center for Vaccine Research and Pandemic Preparedness (C-VaRPP), Icahn School of Medicine at Mount Sinais, New York, NY, United States; ^11^ Ignaz Semmelweis Institute, Interuniversity Institute for Infection Research, Medical University of Vienna, Wien, Austria; ^12^ Department of Medicine, Division of Infectious Diseases, Icahn School of Medicine at Mount Sinai, New York, NY, United States; ^13^ Global Health and Emerging Pathogens Institute, Icahn School of Medicine at Mount Sinai, New York, NY, United States; ^14^ The Tisch Cancer Institute, Icahn School of Medicine at Mount Sinai, New York, NY, United States; ^15^ The Icahn Genomics Institute, Icahn School of Medicine at Mount Sinai, New York, NY, United States; ^16^ GlobalBio, Inc., Cambridge, MA, United States

**Keywords:** COVID-19, Newcastle disease virus-based vaccines, Wuhan strain, Omicron BA.1, Omicron XBB.1.16, Omicron JN.1, neutralizing antibodies, AVX/COVID-12

## Abstract

**Introduction:**

Severe acute respiratory syndrome coronavirus 2 (SARS-CoV-2) remains a global health challenge, causing severe morbidity and mortality, particularly in vulnerable groups such as the elderly, immunocompromised individuals, and those with comorbidities. In low- and middle-income countries (LMICs), vaccine access is hindered by high costs and inequitable distribution. To tackle these issues, Mexico developed the AVX/COVID-12 (V-Wu) vaccine, a recombinant Newcastle disease virus (NDV)-based platform expressing a stabilized ancestral Wuhan spike protein (HexaPro-S). Locally manufactured after rigorous testing and regulatory approval, V-Wu aims to enhance self-sufficiency and equity in immunization.

**Methods:**

This study evaluates an updated vaccine version, AVX/COVID-12 (V-BA), designed to combat Omicron subvariants by expressing the HexaPro-S protein of BA.2.75.2. Both vaccines were administered intramuscularly in K18-hACE2 transgenic and BALB/c mouse models using a prime-boost regimen. Immunogenicity was analyzed by measuring antibodies against Omicron S proteins BA.2.75.2 and XBB.1.5, as well as neutralizing antibodies against Wuhan, BA.1, XBB.1.16, and JN.1 variants.

**Results:**

Both vaccines were safe, eliciting robust antibody responses against Omicron S proteins and neutralizing antibodies against multiple emerging SARS-CoV-2 variants of concern (VOCs). V-BA demonstrated superior protection against current Omicron variants, while V-Wu offered broader coverage, including the ancestral Wuhan strain and emerging variants like JN.1.

**Discussion:**

These findings underscore the adaptability of NDV-based platforms in addressing the evolving SARS-CoV-2 landscape and reaffirm the ongoing utility of the ancestral Patria vaccine. Together, they demonstrate the potential of these platforms to drive the development of next-generation vaccines tailored to emerging viral threats, contributing to global health equity.

## 1 Introduction

Since the beginning of the coronavirus 2019 (COVID-19) pandemic, severe acute respiratory syndrome coronavirus 2 (SARS-CoV-2) and its emerging variants have caused millions of deaths and continue to drive substantial morbidity, particularly among vulnerable populations such as individuals with comorbidities, immunocompromised patients, and the elderly (1). The rapid global spread of the virus has highlighted the critical need for effective vaccination strategies to reduce disease burden and prevent long-term complications. While first-generation vaccines have significantly mitigated the impact of the pandemic, the continual emergence of SARS-CoV-2 variants of concern (VOCs) has compromised their effectiveness, reinforcing the necessity of developing next-generation vaccines as a public health priority (2).

Access to vaccines has presented a significant challenge, particularly in low- and middle-income countries (LMICs), where inequitable distribution and high costs have hindered broad immunization coverage (3–5). In Mexico, these barriers underscored the urgent need to develop and manufacture vaccines locally to achieve self-sufficiency and ensure equitable access for the population. To address this challenge, a multidisciplinary collaboration among the Mexican company AVIMEX, the Icahn School of Medicine at Mount Sinai (USA), Mexican government agencies, and various healthcare and academic institutions led to the development of the AVX/COVID-12 vaccine, known as “Patria” (6–8).

The AVX/COVID-12 vaccine is based on a recombinant NDV-platform and expresses the stabilized prefusion S protein (HexaPro-S) of the ancestral Wuhan-1 SARS-CoV-2 strain (9). Preclinical studies demonstrated its safety and immunogenicity, showing the induction of specific antibody and cellular immune responses, including cross-reactivity with some VOCs (10, 11). Phase I, II, and II/III clinical trials (6–8) further confirmed its safety and immunogenicity when administered via intranasal or intramuscular routes. These findings established AVX/COVID-12 as a promising booster vaccine against the original SARS-CoV-2 strain and led to its regulatory approval for adult use in Mexico (12).

However, the ongoing evolution of SARS-CoV-2 underscores the need to update existing vaccines to maintain their efficacy against emerging VOCs. In this study, we evaluated the original AVX/COVID-12 vaccine alongside a newly developed version engineered to express the S protein of the Omicron BA.2.75.2 sublineage. We discuss the findings and their implications for vaccination strategies using AVX/COVID-12 and its updated formulation to enhance protection against emerging VOCs.

## 2 Materials and methods

### 2.1 Vaccine candidates

The vaccines evaluated in this study were based on a live recombinant NDV vector expressing the S protein of SARS-CoV-2, as previously described (8, 11, 13). The AVX/COVID-12 vaccine (V-Wu) expresses the S protein of the ancestral Wuhan-1 strain, while the updated NDV-based vaccine (V-BA) displays the S protein of the SARS-CoV-2 Omicron sublineage BA.2.75.2. In both constructs, the S protein includes six proline substitutions and a deletion of the polybasic cleavage site to stabilize it in the prefusion conformation. Additionally, the ectodomain of the S protein is fused to the transmembrane and cytoplasmic domains of the NDV fusion protein to promote optimal incorporation into the viral particle. Vaccine production was carried out by Laboratorio Avi-Mex, S.A. de C.V. in Mexico City using embryonated eggs, under good manufacturing practices (GMP), as previously reported (8, 11, 13).

### 2.2 Animal models and experimental design

**Figure 1:**
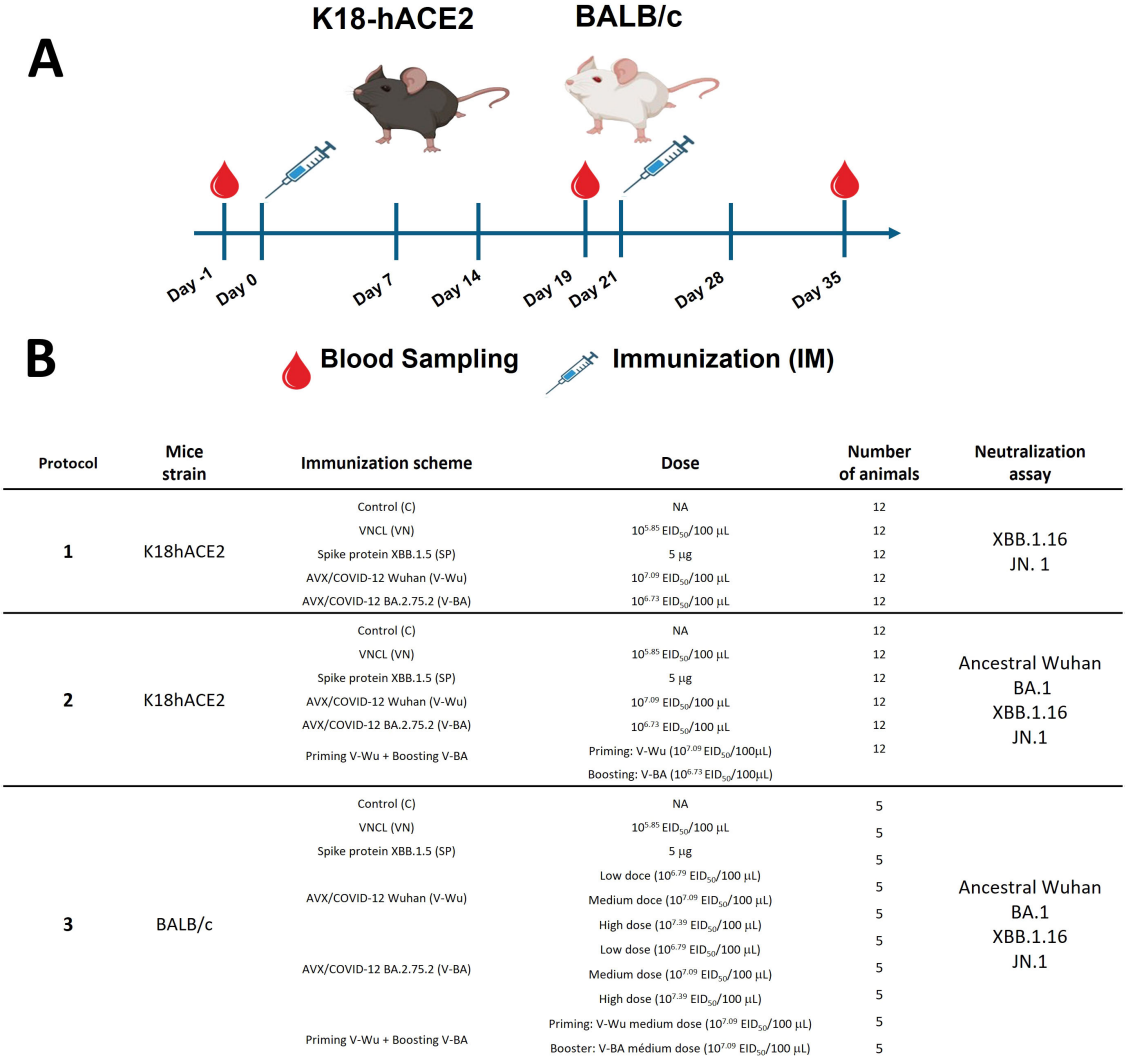
Schematic experimental design. Two mouse models were immunized intramuscularly (IM) with the experimental vaccines AVX/COVID-12 Wuhan (V-Wu) and BA.2.75.2 (V-BA). **(A)** Mice were vaccinated on days 0 and 21. Blood samples were collected on days -1, 19, and 35 via submandibular puncture. **(B)** The table illustrates the different groups divided into three experimental protocols. Protocols 1 and 2 utilized K18-hACE2 mice with a homologous prime-boost immunization scheme, except for group 6 in protocol 2, which received a heterologous prime-boost with V-Wu on day 0 and V-BA on day 21. Protocol 3 analyzed immunization schemes in BALB/c mice, including additional modifications with low (L), medium (M), and high (H) doses of V-Wu and V-BA in a homologous scheme.

This study assessed the safety and immunogenicity of two experimental vaccines using K18-human angiotensin-converting enzyme 2 (hACE2) transgenic mice and BALB/c mice, as illustrated in **Figure 1**. Transgenic mice were obtained from The Jackson Laboratory (Bar Harbor, ME, USA), while BALB/c mice were bred at the animal facility of the Unidad de Desarrollo e Investigación en Bioterapéuticos (UDIBI). Mice were immunized intramuscularly on day 0, followed by a booster dose on day 21. Blood samples were collected at three time points: baseline titers were measured on day -1 (D-1), pre-booster samples were taken on day 19 (D19), and post-booster samples were collected two weeks later, on day 35 (D35).

Safety assessment: Body weight variation throughout the study was used as a key indicator of the animals’ health, expressed as a percentage relative to each animal’s weight on day 0. Additionally, skin condition, hair loss, nasal moisture, behavior, and posture were monitored three times per week. All procedures involving animals complied with national regulations (NOM-062-ZOO-1999) and the guidelines of the Office of Laboratory Animal Welfare (OLAW) of the National Institutes of Health (NIH). Mice were housed in standard cages under controlled temperature and humidity, with a 12-hour light/dark cycle.

### 2.3 Isolation and identification of SARS-CoV-2 Omicron sublineages

SARS-CoV-2 samples were collected during the winter of 2023–2024 from individuals presenting typical COVID-19 symptoms and confirmed positive by reverse transcription polymerase chain reaction (RT-PCR), as previously described by González-González et al. (14). Nasopharyngeal swabs with the lowest cycle threshold (Ct) values (<25) were stored in Eagle’s Minimum Essential Medium (EMEM; Manassas, VA, USA; Cat. No. 30-2003). Sample collection complied with the principles of the Declaration of Helsinki (15), with informed consent obtained from all participants. All virus handling was conducted under BSL-2+ conditions in accordance with biosafety guidelines from the World Health Organization (WHO) and the U.S. Centers for Disease Control and Prevention (CDC) (16–18).

Virus isolation: SARS-CoV-2 propagation was performed in Vero cells (ATCC, Cat. No. CCL-81). Cells were incubated with viral samples for 60 hours, followed by a 24-hour freezing period to induce cell lysis. Cellular debris was removed by centrifugation, and supernatants were collected and titrated using a plaque assay (19). RNA was extracted from culture supernatants with the highest viral titers using the MagMAX™ Viral and Pathogen Nucleic Acid Isolation Kit (Applied Biosystems™, Thermo Fisher Scientific, Austin, TX, USA; Cat. No. A42352), according to the manufacturer’s instructions.

Sanger sequencing of the SARS-CoV-2 S1 gene segment: Primary screening to identify Omicron sublineages XBB.1.16.15 and JN.1 was performed by Sanger sequencing. cDNA was synthesized from RNA samples using the ProtoScript II First Strand cDNA Synthesis Kit (New England Biolabs, Ipswich, MA, USA). A fragment encoding the S1 subunit (nucleotides 950–1945, amino acids 325–642) was amplified using the forward primer 5′-ACTTTAGAGTCCAACCAACAGAA-3′ and the reverse primer 5′-AGCCTGCACGTGTTTGAAAA-3′. PCR amplification was conducted using Phusion Hot Start Flex DNA Polymerase (New England Biolabs, USA), and product quality and size were confirmed by 1% agarose gel electrophoresis. PCR products were purified using the QIAquick PCR Purification Kit (Qiagen, Germantown, MD, USA) and submitted for Sanger sequencing at Wyzer Biosciences, Inc. (Cambridge, MA, USA).

SARS-CoV-2 whole-genome sequencing: RNA samples containing mutations corresponding to Omicron sublineages XBB.1.16.15 and JN.1 were submitted for whole-genome sequencing at the Massive Sequencing and Bioinformatics University Unit (UUSMB) of the National Autonomous University of Mexico (UNAM). Sequencing was performed using the ARTIC v5.3.2 COVID-19 Illumina library preparation protocol and the V.5 sequencing protocol (20). Sequencing was conducted on a NextSeq500 platform (Illumina, San Diego, CA, USA) with paired-end reads (2 × 150 bp).

Resulting sequences were genotyped using the Pangolin web server (21, 22). All genomes achieved ≥99% coverage and a mean depth of ≥1000X. Sequences were deposited in GenBank under the following accession numbers: PP837785.1 (isolate AJ153, Omicron XBB.1.16.15), PP837746.1 (isolate AJ221, Omicron BA.1.86), and JN.1. Additionally, this study included the SARS-CoV-2 ancestral Wuhan-1 strain and the Omicron BA.1 subvariant, both previously isolated in our laboratory and reported by González-González et al. (14). Genome sequences for these isolates were deposited in GenBank under accession numbers OL790194 (SARS-CoV-2 Wuhan-1) and ON651664 (SARS-CoV-2 Omicron BA.1).

### 2.4 Enzyme-linked immunosorbent assay to determine specific IgG against the S protein

NUNC MaxiSorp 96-well flat-bottom ELISA plates (Thermo Scientific, Rochester, NY, USA; Cat. No. 456537) were coated overnight at 2–8°C with either SARS-CoV-2 XBB.1.5 S Trimer Protein, His-tagged (Acro Biosystems, Basel, Switzerland; Cat. No. SPN-C524i)—which was also used as an immunogen mixed 1:1 (v/v) with incomplete Freund’s adjuvant (IFA; Sigma-Aldrich)—or SARS-CoV-2 BA.2.75.2 S Trimer Protein, His-tagged (Acro Biosystems; Cat. No. SPN-C522r), both at a concentration of 2 µg/mL in coating buffer (BioRad, Berkeley, CA, USA; Cat. No. BUF030C).

Plates were washed with phosphate-buffered saline containing 0.1% Tween 20 (PBS-T) (PBS 10X, Gibco, Grand Island, NY, USA; Cat. No. 70011-044; Tween 20, Sigma, Darmstadt, Germany; Cat. No. SLCG3047) and then blocked with 3% skim milk in PBS-T for 1 hour at room temperature. Subsequently, inactivated serum samples and controls, diluted in 1% milk-PBS-T, were added to the plates and incubated for 1 hour at room-temperature. Following additional washes with PBS-T, IgG antibodies were detected using horseradish peroxidase (HRP)-conjugated anti-mouse IgG antibody (Invitrogen, Camarillo, CA, USA; Cat. No. 61-6520). Plates were developed with 3,3′,5,5′-tetramethylbenzidine (TMB) substrate (BD OptEIA, San Diego, CA, USA; Cat. No. 555214), and the colorimetric reaction was stopped by adding TMB stop solution (Abcam, Waltham, MA, USA; Cat. No. ab171529). Optical density (OD) was measured at 450 nm with a correction at 570 nm using a SpectraMax M3 microplate reader (Molecular Devices, San Jose, CA, USA).

End-point titers were reported as ELISA units (EU/mL). The quantification range with linear behavior was established between OD values of 0.2–1.4 (450/570 nm), based on a standard curve generated using a positive serum. The lower limit of quantification was defined as the reciprocal of the serum dilution corresponding to an OD value of 0.2 by interpolation, which was also used as the cutoff for a positive response.

### 2.5 Microneutralization assay

Neutralizing antibodies against SARS-CoV-2 were evaluated using a modified microneutralization assay based on the methods described by Amanat et al. (23, 24) and the cell viability protocol reported by Feoktistova et al. (24). Heat-inactivated serum samples were serially diluted and incubated with a fixed amount of live virus (either the ancestral strain or Omicron sublineages). After co-incubation with Vero cells, neutralization was defined as the ability of serum to inhibit virus-induced cytopathic effect (CPE), quantified by crystal violet staining. The 50% inhibitory dilution (ID_50_) was calculated using nonlinear regression analysis.

Microneutralization using authentic SARS-CoV-2 was selected over pseudovirus-based assays to provide a more biologically relevant assessment of viral replication and entry, including the impact of non-spike mutations. While pseudovirus assays offer advantages in throughput and biosafety, live-virus neutralization reflects the complete viral phenotype, offering improved resolution for detecting subtle differences in neutralizing capacity, especially when comparing closely related variants.

## 3 Results

### 3.1 Body weight monitoring and safety evaluation in immunized mice

**Figure 2:**
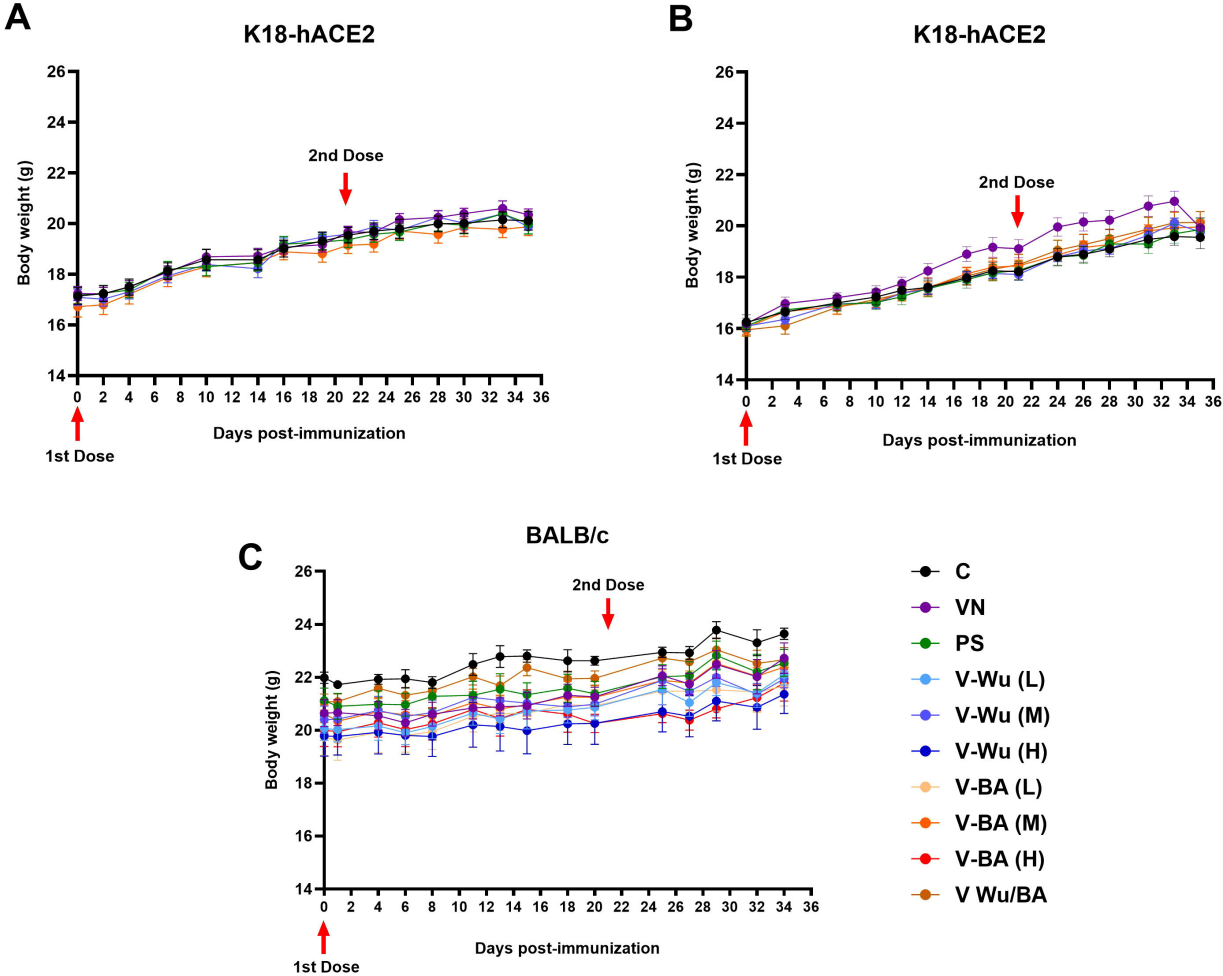
Safety and tolerability of V-Wu and V-BA vaccines post-immunization. Kinetics of body weight in mice following immunization across different experimental protocols. **(A)** K18-hACE2 mice immunized with homologous schemes. **(B)** K18-hACE2 mice immunized with homologous or heterologous schemes (prime with V-Wu and boost with V-BA). **(C)** BALB/c mice immunized with homologous schemes, including variations with low (10^6.79^ EID50/100 µL), medium (10^7.09^ EID50/100 µL), and high (10^7.39^ EID50/100 µL) doses of V-Wu or V-BA, as well as a heterologous scheme (prime with V-Wu at medium dose and boost with V-BA at medium dose). The graphs depict the mean ± SEM of body weight (g) for each study protocol. Red arrows indicate vaccination days for priming (Day 0) and boosting (Day 21). C = Negative control, VN = Vector control, SP = S protein (XBB.1.5 sublineage), V-Wu = AVX/COVID-12 Wuhan vaccine, V-BA = AVX/COVID-12 BA.2.75.2 vaccine, L = Low dose, M = Medium dose, H = High dose, V-Wu/BA = Prime with V-Wu and boost with V-BA. K18-hACE2 groups: n=12 per group; BALB/c groups: n=5 per group. Data were analyzed with two-way ANOVA and Tukey’s *post hoc* test using GraphPad Prism software. Statistical differences are indicated where p < 0.05.

To assess the safety of the V-Wu and V-BA vaccines, weight variations were monitored in all experimental groups throughout the study. In K18-hACE2 mice, no significant differences in weight gain were observed, with trends comparable to those of the control group (**Figures 2A, B**). Similarly, BALB/c mice showed no significant changes in weight gain (**Figure 2C**). Within-group analysis showed that BALB/c mice gained weight more slowly than K18-hACE2 mice, a difference attributable to strain-specific factors, including age and initial body weight. At study initiation, K18-hACE2 mice weighed 16–18 g and were six weeks old, whereas BALB/c mice weighed 19–22 g and were seven weeks old. Despite these initial differences, neither vaccine had a negative impact on normal growth or weight gain in either strain, as confirmed by comparisons with the unvaccinated control group. Additional clinical observations including assessments of skin condition, coat quality, nasal moisture, and behavior, revealed no abnormalities in any group during the study.

### 3.2 V-Wu and V-BA immunization induced specific antibodies against Omicron BA.2.75.2 and XBB.1.5 sublineages

**Figure 3:**
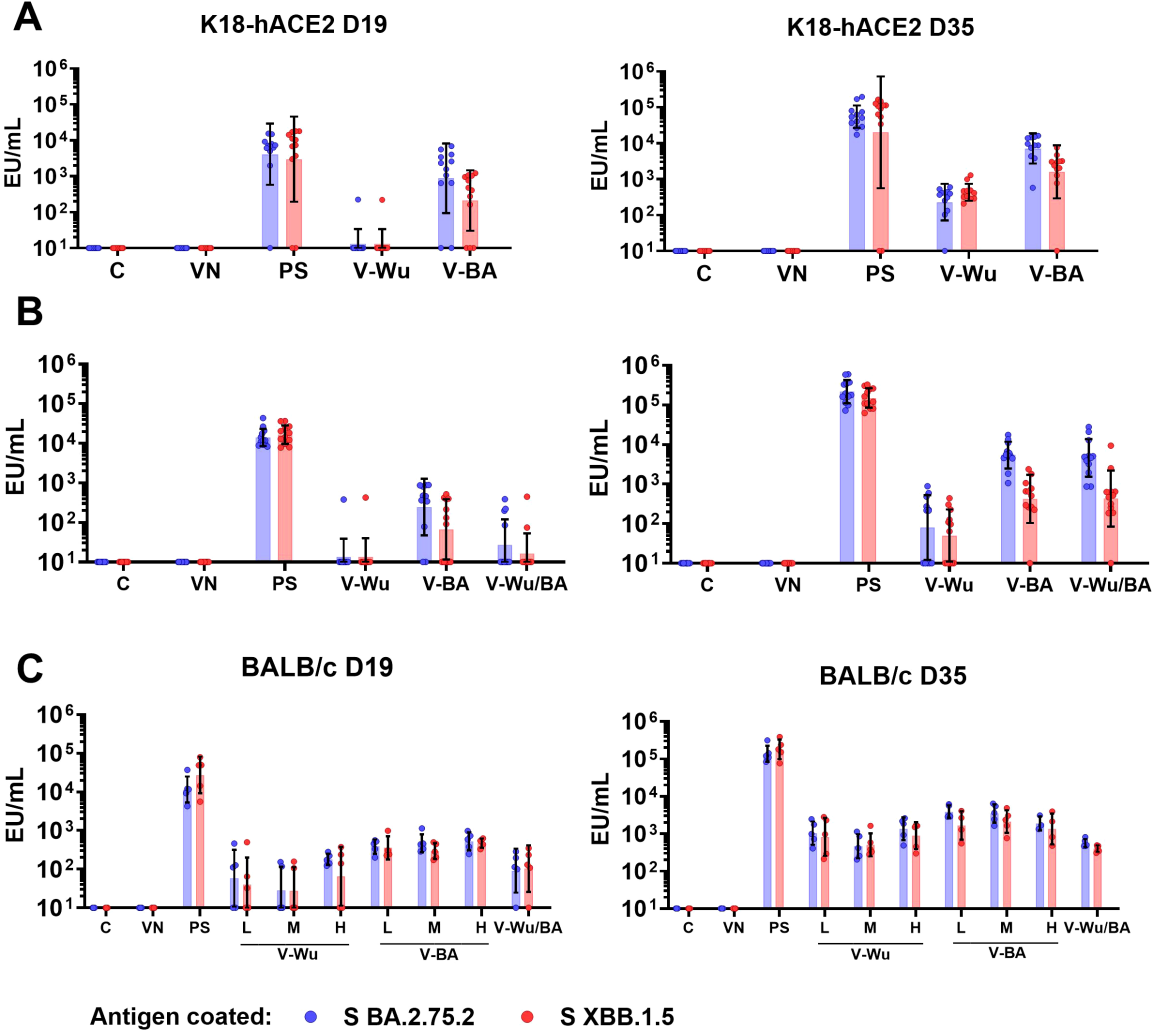
Immunization with V-Wu and V-BA induces specific antibodies against BA.2.75.2 and XBB.1.5 S proteins. Specific IgG antibodies expressed as ELISA units (EU) per mL against the S proteins of SARS-CoV-2 BA.2.75.2 and XBB.1.5 variants were measured by ELISA on days 19 and 35 post-immunization. **(A)** K18-hACE2 mice immunized with homologous schemes. **(B)** K18-hACE2 mice immunized with homologous and heterologous schemes (priming with V-Wu and boosting with V-BA). **(C)** BALB/c mice immunized with homologous schemes, including variations in dose (low: 10^6.79^ EID50/100 µL, medium: 10^7.09^ EID50/100 µL, and high: 10^7.39^ EID50/100 µL) for V-Wu or V-BA, as well as a heterologous scheme (priming with V-Wu at medium dose and boosting with V-BA at medium dose). The graphs display the geometric mean ± SD of EU/mL for each group. C = Negative control, VN = Vector control, SP = S protein (XBB.1.5 sublineage), V-Wu = AVX/COVID-12 Wuhan vaccine, V-BA = AVX/COVID-12 BA.2.75.2 vaccine, L = Low dose, M = Medium dose, H = High dose, V-Wu/BA = Prime with V-Wu and boost with V-BA. Sample sizes: K18-hACE2 groups (n = 12 per group); BALB/c groups (n = 5 per group).

The antibody responses induced by homologous immunization with either V-Wu or V-BA were evaluated in K18-hACE2 mice against the S proteins of Omicron sublineages BA.2.75.2 and XBB.1.5. No significant differences in antibody titers were observed following the first dose with V-Wu (**Figures 3A, B**). However, by day 35, a homologous booster dose with either V-Wu or V-BA increased antibody titers compared to control or empty vector groups (**Figures 3A, B**). In the BALB/c mouse model, the effect of vaccine dose (low, medium, or high) on the primary antibody response was assessed for both V-Wu and V-BA. Antibody titers observed on days 19 and 35 were comparable to those obtained in K18-hACE2 mice, with no significant differences between dosage levels. When comparing the heterologous regimen of V-Wu priming followed by V-BA boosting to the homologous V-BA regimen in K18-hACE2 mice, both approaches induced similar antibody titers (**Figure 3B**). However, in BALB/c mice, the heterologous vaccination elicited lower titers, with levels remaining comparable between the priming and booster dose (**Figure 3C**). Overall, both homologous and heterologous vaccination regimens using either V-Wu or V-BA induced specific antibody responses against the Omicron sublineages BA.2.75.2 and XBB.1.5, with comparable titers across groups.

### 3.3 V-Wu and V-BA induced neutralizing antibody responses against the ancestral strain and Omicron sublineages

**Figure 4:**
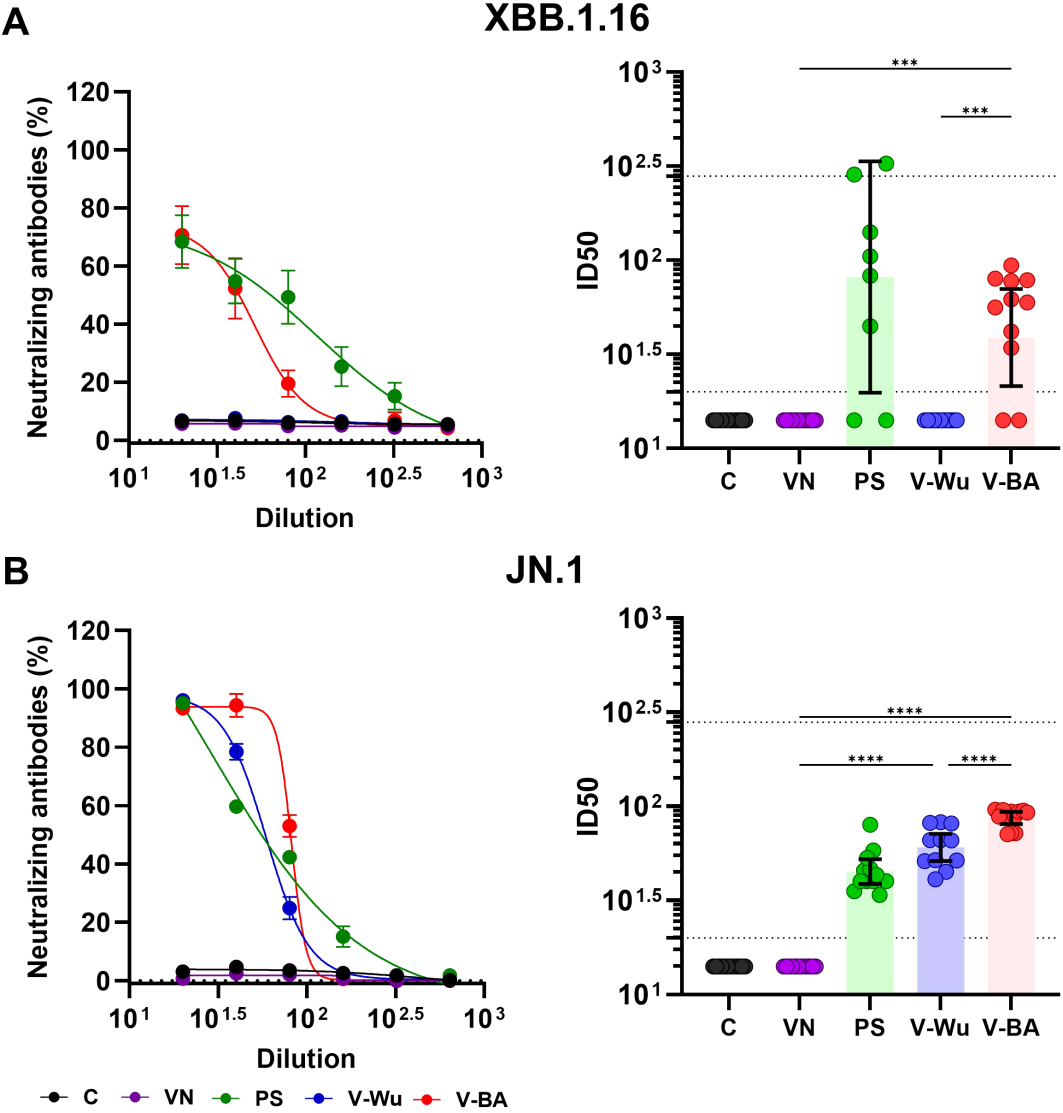
Neutralizing antibody responses against XBB.1.16 and JN.1 Omicron variants induced by homologous immunization with V- Wu or V-BA vaccines. Neutralizing activity of sera from K18-hACE2 mice immunized with homologous vaccine schemes was assessed against SARS-CoV-2 variants **(A)** XBB.1.16 or **(B)** JN.1 Omicron sublineages. The left panels display the neutralization percentage (mean ± SEM) derived from serial dilutions of sera collected on day 35 post-boost. The right panels show the ID50 values (mean ± 95% CI) calculated at the same time point. Dotted lines indicate the assay’s detection limit (lower line) and the response level of the positive control (upper line). Groups: C = Negative control, VN = Vector control, SP = S protein (XBB.1.5 sublineage), V-Wu = AVX/COVID-12 Wuhan vaccine, V-BA = AVX/COVID-12 BA.2.75.2 vaccine. Sample size: n = 12 per group. Data were analyzed with one-way ANOVA and Tukey’s *post hoc* test using GraphPad Prism software. Statistical significance is denoted as ****p < 0.0001 and ***p < 0.001.

Both vaccines induced comparable overall antibody titers, suggesting similar levels of immune activation. However, differences were observed in their neutralizing capacity against Omicron sublineages. Neutralizing antibody titers were evaluated on day 35 following homologous vaccination with V-Wu or V-BA, as illustrated by dilution curves and ID_50_ values in K18-hACE2 mice (**Figure 4**). V-Wu failed to induce neutralizing antibodies against the XBB.1.16 strain but elicited a statistically significant response against the JN.1 variant compared to control groups (**Figure 4A**).

In contrast, V-BA vaccination led to a statistically significant increase in neutralizing antibodies against both XBB.1.16 and JN.1 sublineages compared to V-Wu (**Figure 4B**). No neutralizing activity was detected in the control or empty vector groups. Immunization with recombinant XBB.1.5 S protein served as a positive control, inducing responses against both Omicron sublineages.

**Figure 5:**
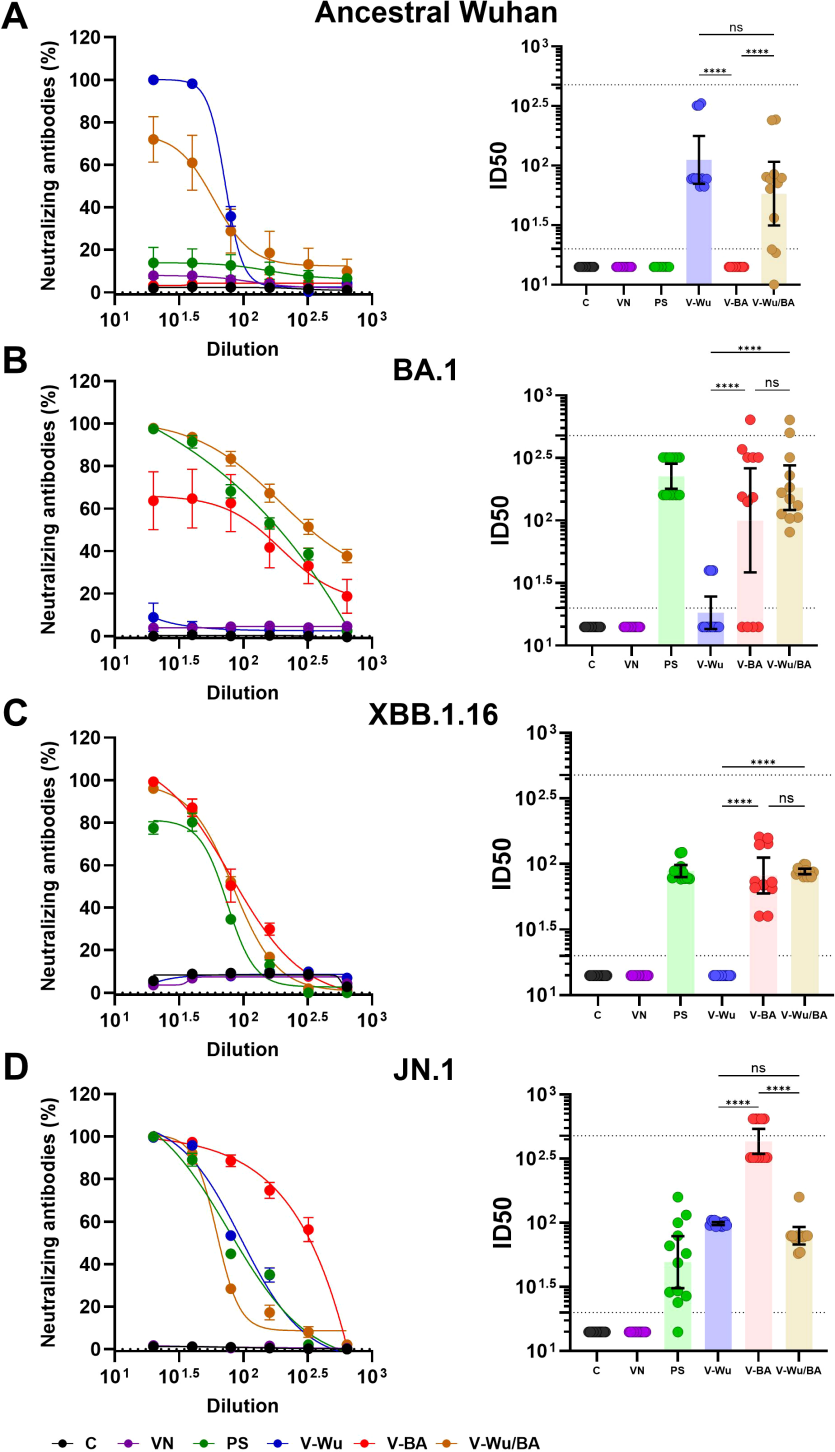
Neutralizing antibody responses against ancestral Wuhan and Omicron variants induced by homologous and heterologous immunization with V-Wu or V-BA vaccines. Neutralizing activity of sera from K18-hACE2 mice immunized with homologous (V-Wu/Wu or V-BA/BA) or heterologous (V-Wu/BA) vaccine schemes was evaluated against SARS-CoV-2 variants: **(A)** ancestral Wuhan-1, **(B)** BA.1, **(C)** XBB.1.16, and **(D)** JN.1 Omicron sublineages. The left panels illustrate neutralization percentages (mean ± SEM) obtained from serial dilutions of sera collected on day 35 post-boost. The right panels present ID50 values (mean ± 95% CI) calculated for the same time point. Dotted lines represent the assay’s detection limit (lower line) and the response level of the positive control (upper line). Groups: C = Negative control, VN = Vector control, SP = S protein (XBB.1.5 sublineage), V-Wu = AVX/COVID-12 Wuhan vaccine, V-BA = AVX/COVID-12 BA.2.75.2 vaccine, V-Wu/BA = Prime with V-Wu and boost with V-BA. Sample size: n = 12 per group. Data were analyzed with one-way ANOVA and Tukey’s *post hoc* test using GraphPad Prism software. Statistical significance is denoted as ****p < 0.0001, and ns = not significant.

Homologous and heterologous vaccination schemes were further compared (**Figure 5**). V-Wu elicited significantly higher neutralizing antibodies against the ancestral strain compared to V-BA (**Figure 5A**), in line with earlier observations (**Figure 4**). V-Wu also induced neutralizing antibodies against JN.1 but not against XBB.1.16, and exhibited a weak and variable response against the BA.1 sublineage (**Figures 5B–D**). In contrast, V-BA induced neutralizing antibodies against Omicron sublineages BA.1, XBB.1.16, and JN.1, but not against the ancestral strain (**Figure 5**). The heterologous regimen elicited neutralizing antibodies against the ancestral strain as well as Omicron sublineages BA.1, XBB.1.16, and JN.1.

**Figure 6:**
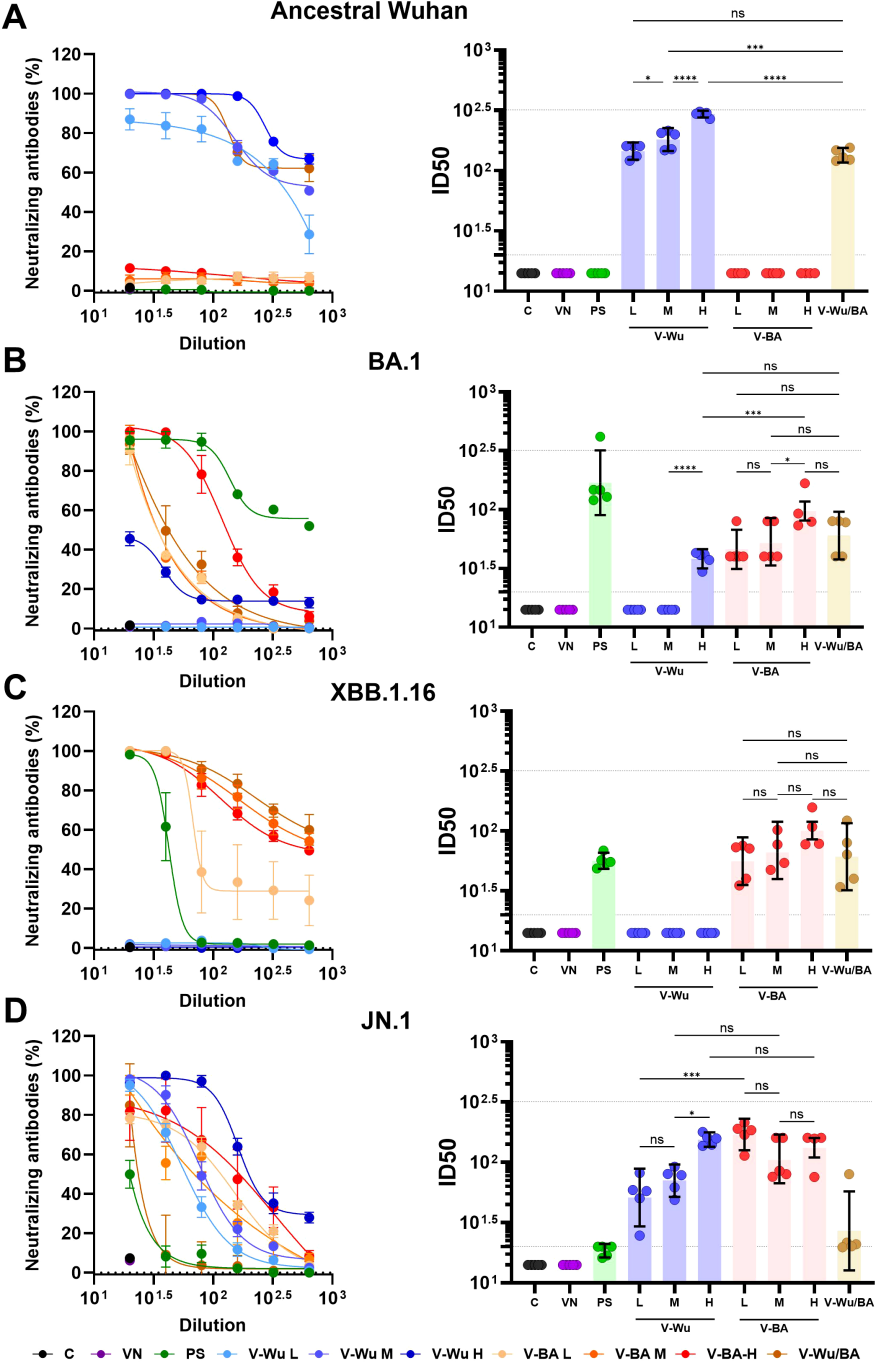
Neutralizing antibody responses against ancestral Wuhan and Omicron variants induced by dose-response homologous or heterologous immunization with V-Wu or V-BA vaccines. Neutralizing activity of sera from BALB/c mice immunized with homologous (V-Wu/Wu or V-BA/BA) schemes, including variations in dose (low: 10^6.79^ EID50/100 µL, medium: 10^7.09^ EID50/100 µL, and high: 10^7.39^ EID50/100 µL), or heterologous (V-Wu/BA) vaccine schemes was assessed against SARS-CoV-2 variants: **(A)** ancestral Wuhan-1, **(B)** BA.1, **(C)** XBB.1.16, and **(D)** JN.1 Omicron sublineages. The left panels display neutralization percentages (mean ± SEM) derived from serial dilutions of sera collected on day 35 post-boost, while the right panels present ID50 values (mean ± 95% CI) calculated for the same time point. Dotted lines indicate the assay’s detection limit (lower line) and the response level of the positive control (upper line). C = Negative control, VN = Vector control, SP = S protein (XBB.1.5 variant), V-Wu = AVX/COVID-12 Wuhan vaccine, V-BA = AVX/COVID-12 BA.2.75.2 vaccine, L = Low dose, M = Medium dose, H = High dose, V-Wu/BA = Prime with V-Wu and boost with V-BA. Sample size: n = 5 per group. Data were analyzed with one-way ANOVA and Tukey’s *post hoc* test using GraphPad Prism software. Statistical significance is denoted as ****p < 0.0001, ***p < 0.001, *p < 0.05 and ns = not significant.

In BALB/c mice, neutralizing responses were consistent with those observed in K18-hACE2 mice (**Figure 6**). V-Wu induced dose-dependent neutralizing antibodies against both the ancestral strain and JN.1 variant (**Figures 6A, D**). It also induced neutralization antibodies against BA.1, but not against XBB.1.16 (**Figures 6B, C**). Notably, the high-dose V-Wu group exhibited neutralizing response comparable to those of the low- and medium-dose V-BA groups. Heterologous vaccination induced neutralizing antibodies against all SARS-CoV-2 strains tested (**Figure 6**), supporting its potential to broaden protection against diverse variants included in this study.

## 4 Discussion

The V-Wu vaccine, based on the ancestral Wuhan strain, has demonstrated safety, tolerability, and immunogenicity in phase 1–3 clinical trials (6–8). Furthermore, a non-inferiority trial showed that the antibody responses elicited by V-Wu were comparable to those induced by the ChAdOx1-S vaccine from AstraZeneca (6). These findings supported its regulatory approval for use as a booster vaccine in Mexico (12). However, the ongoing and rapid evolution of SARS-CoV-2 has prompted vaccine developers to update formulations targeting prevalent variants. Unfortunately, the pace of viral evolution continues to outstrip the ability to design, produce, and evaluate variant-specific vaccines, highlighting the need for next-generation formulations capable of inducing broad and durable immune responses against multiple circulating variants.

The spike protein used in both V-Wu and V-BA vaccines incorporates the HexaPro stabilization strategy, which consists of six proline substitutions that preserve the protein in its prefusion conformation. This modification improves the structural stability of the spike trimer, enhances expression, and increases immunogenicity by maintaining key neutralizing epitopes in their native configuration (9).

In this context, the development of the V-BA vaccine aimed to target broader conserved epitopes shared among diverse circulating SARS-CoV-2 variants. The BA.2.75.2 sublineage was selected as the antigenic basis for this formulation, as it represents a close common ancestor of many currently circulating strains (19). The results from the preclinical mouse model described in this study demonstrated that V-BA is safe and well-tolerated, with a safety profile comparable to that of V-Wu. Both K18-hACE2 and BALB/c mice showed favorable responses, with no observable adverse effects on general health status.

In Mexico, most available COVID-19 vaccines, including V-Wu, are based on the ancestral Wuhan strain, and the majority of the population has been immunized exclusively with these first-generation formulations. To simulate a potential real-world application of V-BA in a previously vaccinated population, we evaluated the immune response induced by a heterologous regimen consisting of V-Wu priming followed by V-BA boosting. Our findings confirmed that this combination was also safe and well-tolerated in both animal models.

Furthermore, the V-BA vaccine induced robust antibody responses in both K18-hACE2 and BALB/c mice, comparable to those induced by V-Wu, demonstrating its high immunogenicity. Notably, V-BA elicited neutralizing antibodies against the Omicron sublineages BA.2.75.2, XBB.1.5, and JN.1, but failed to generate a neutralizing response against the ancestral SARS-CoV-2 strain. This lack of cross-neutralization may be attributed to the substantial genetic divergence between Omicron sublineages and the ancestral Wuhan strain.

**Figure 7:**
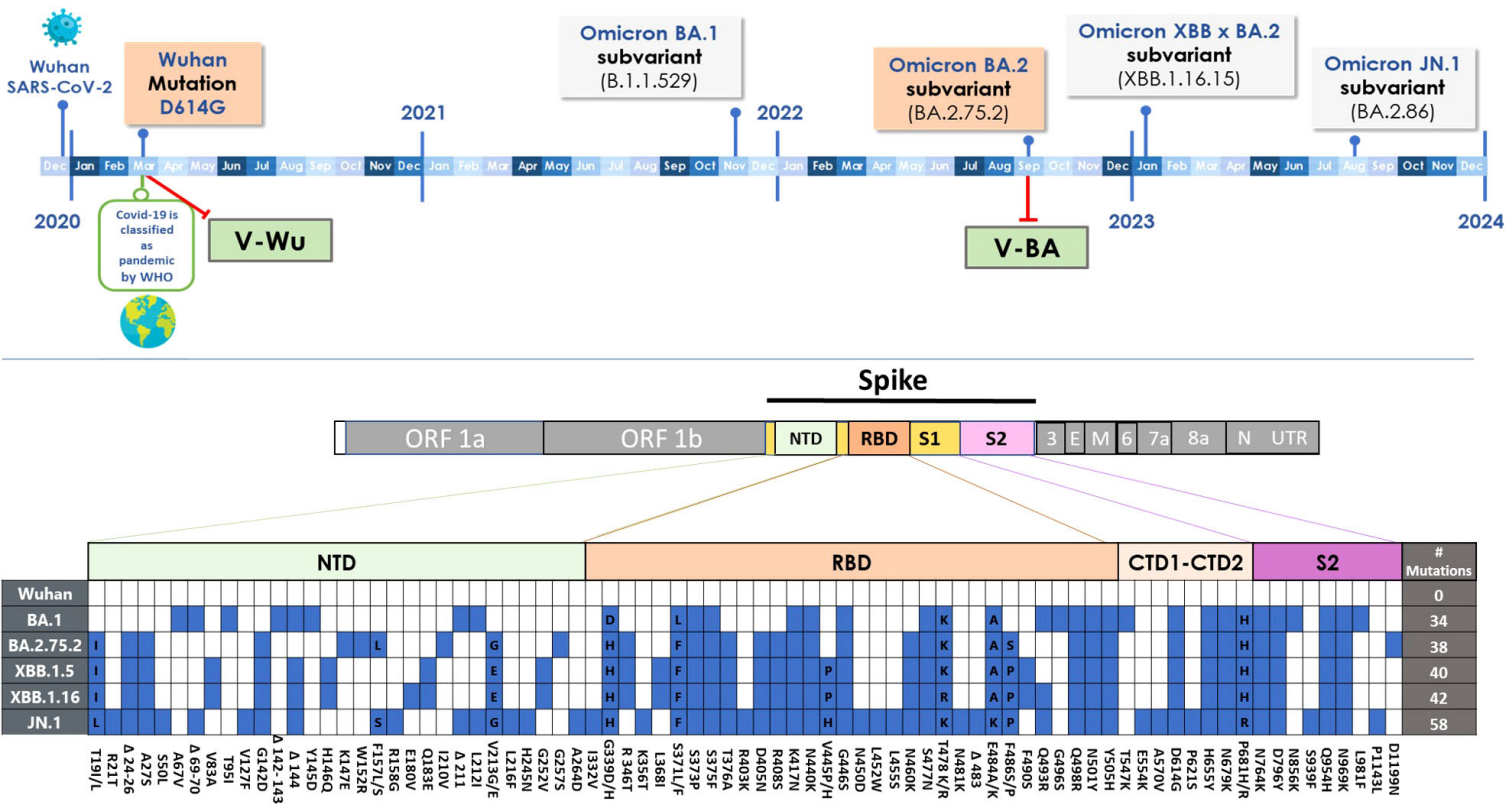
SARS-CoV-2 evolution. Upper panel: Timeline illustrating the emergence of SARS-CoV-2 and Omicron variants. Variants of the S protein incorporated into vaccine designs are highlighted in red boxes, while additional S protein variants used in ELISA or neutralization assays are shown in grey boxes. Lower panel: Mutations (blue squares) in the S protein of Omicron subvariants relative to the ancestral Wuhan-1 virus.

The first Omicron sublineage (BA.1), identified in November 2021 (25), represented the most antigenically distinct variant reported to date, with major immune escape properties. BA.1 carried five deletions and 34 mutations in the spike protein (26), including 15 within the receptor-binding domain (RBD) (as illustrated in the lower panel of **Figure 7**), significantly altering its antigenic profile. As a result, most prophylactic and therapeutic monoclonal antibodies previously authorized by the U.S. Food and Drug Administration for COVID-19 treatment lost efficacy against BA.1 (27). Therefore, it is not surprising that a vaccine based on the Omicron BA.2.75.2 S protein did not induce neutralizing antibodies against the ancestral Wuhan strain.

The observation that V-Wu elicited specific antibodies against the S protein of Omicron BA.2.75.2 and XBB.1.5, both after homologous boosting and heterologous boosting with V-BA, may be explained by their evolutionary relationship to the BA.1 and XBB.1.16 sublineages (25, 26). Interestingly, V-Wu induced a strong neutralizing response against JN.1, yet only weak titers against BA.1 and no response against XBB.1.16. This differential activity may be influenced by several factors, including antigen specificity, the presence of revertant mutations in JN.1 that may enhance recognition by antibodies induced by the ancestral Wuhan strain, and differences in vaccine dose or individual immune responsiveness. Additionally, structural differences between variants may play a critical role: for instance, the L455S mutation in the S protein of XBB.1.16 disrupts a hydrophobic cavity essential for binding IGHV3-53/3–66 class neutralizing antibodies, contributing to immune escape (26, 28). This mutation is absent or less prevalent in JN.1, potentially allowing for greater cross-recognition. Moreover, the extensive accumulation of RBD mutations in XBB.1.16 may reduce epitope accessibility for V-Wu-induced antibodies, whereas JN.1 may retain structural elements more similar to earlier strains (26, 28). These findings highlight the challenge posed by the continuous antigenic evolution of SARS-CoV-2 and underscore the need for ongoing surveillance and antigenic characterization of emerging variants to guide future vaccine development.

Another possible explanation for the observed cross-reactivity lies in the structural modifications of the S protein, which enhance both its stability and immunogenic potential, as previously reported (9, 29). Both vaccines are based on an NDV platform incorporating HexaPro stabilization, a strategy that maintains class I fusion proteins in their prefusion conformation (9). This approach has proven essential for exposing a broader range of epitopes in both open and closed conformations, thereby improving the breadth and potency of neutralizing responses (9, 29). The efficacy of similar stabilization techniques is exemplified by recently approved vaccines against respiratory syncytial virus (RSV) (30). Furthermore, advanced stabilization strategies, such as disulfide bond introduction and cavity-filling substitutions, have been successfully applied to vaccine candidates targeting RSV, ebolavirus, influenza virus, and human immunodeficiency virus type 1 (HIV-1) (30–33).

Building on these technological advancements, the V-Wu and V-BA vaccines demonstrate a strong capacity to elicit broad immunogenic responses, underscoring their potential to combat emerging SARS-CoV-2 variants. Preclinical and clinical trials with V-Wu have shown that it induces high antibody titers and T-cell activation against both the ancestral strain and several VOCs following primary immunization and/or booster administration (7, 8, 11). *In vitro* antigenicity assays using V-Wu have further demonstrated its ability to restimulate cellular responses, with T-cells recognizing conserved epitopes encoded by the vaccine in individuals who had recovered from COVID-19 or were previously vaccinated with other platforms (13). Moreover, these epitopes exhibited cross-reactivity with antibodies generated in response to the ancestral Wuhan strain and early Omicron variants, highlighting the presence of conserved B- and T-cell epitopes across different SARS-CoV-2 lineages (13, 34).

Our findings have important implications for the development of next-generation vaccines and the design of vaccination strategies. First, both priming and boosting with V-Wu elicited a robust neutralizing response against the ancestral strain as well as recent SARS-CoV-2 VOCs, particularly JN.1 and BA.1 (especially at higher doses). Given the rapid evolution of SARS-CoV-2 and the time and resources required to develop and evaluate updated vaccines, these results suggest that V-Wu, already validated in clinical trials, could serve as an effective booster to provide protection against recent VOCs. Second, increasing the vaccine dose may further enhance the breadth of the neutralizing response. Third, heterologous vaccination was shown to induce neutralizing antibodies against all SARS-CoV-2 variants tested. Thus, a priming dose with V-Wu followed by a V-BA booster may offer additional advantages by enhancing and broadening antibody responses against emerging VOCs.

NDV-based vaccines offer several important advantages that make them particularly well-suited for use in LMICs. Unlike mRNA vaccines, which require ultra-cold storage and complex manufacturing infrastructure, NDV-based vaccines can be produced at low cost using embryonated chicken eggs a method already established for seasonal influenza vaccines. This platform enables local manufacturing in facilities with existing egg-based capacity, promoting regional vaccine sovereignty and reducing reliance on global supply chains (35). Additionally, NDV formulations whether lyophilized or in liquid form are stable under standard refrigeration, simplifying storage and distribution in remote or underserved regions. These logistical and economic benefits make NDV a highly adaptable and scalable vaccine platform for LMIC settings.

Beyond production and distribution advantages, NDV-based vaccines also show promise for repeated administration. NDV is not a common human pathogen, and pre-existing immunity in the general population is rare. While theoretical concerns exist about anti-vector immunity limiting booster efficacy, data from phase I clinical trials with V-Wu demonstrated minimal seroconversion against NDV after a single dose, and only low and transient antibody responses were detected even after three doses (phase II) (7). These findings suggest that repeated NDV-based vaccination may be feasible without significantly compromising immunogenicity (35).

In conclusion, this study demonstrates that V-BA is safe, well-tolerated, and immunogenic in mice, eliciting strong and broad neutralizing antibody responses against multiple Omicron sublineages. These responses were observed in both homologous and heterologous regimens, with the latter combining V-Wu priming followed by V-BA boosting. Together, these results highlight the versatility of the NDV platform to adapt to the evolving SARS-CoV-2 landscape and reaffirm the relevance of the ancestral Patria vaccine. Collectively, our findings support the continued development of NDV-based vaccines as flexible, affordable, and scalable tools for next-generation immunization strategies, particularly in resource-limited settings where accessibility and infrastructure pose challenges to advanced platforms such as mRNA.

## 5 Limitations

This study has several limitations that should be acknowledged. First, the experiments were conducted in small animal models and with limited sample sizes, which may restrict the generalizability of the findings. Second, although we evaluated humoral responses in detail, cellular immune responses particularly T-cell activation and memory were not assessed in this preclinical study. Such data are critical to understanding long-term protection and cross-variant immunity. Third, no viral challenge experiments were performed to evaluate *in vivo* protection against infection or disease. Future studies should include detailed T-cell profiling and viral challenge models to further characterize the protective efficacy and durability of immune responses induced by V-Wu and V-BA.

## Data Availability

The datasets presented in this study can be found in online repositories. The names of the repository/repositories and accession number(s) can be found in the article/supplementary material.
